# Editorial: Turning the Mind's Eye Inward: The Interplay Between Selective Attention and Working Memory

**DOI:** 10.3389/fnhum.2015.00616

**Published:** 2015-11-10

**Authors:** Elger Abrahamse, Steve Majerus, Wim Fias, Jean-Philippe van Dijck

**Affiliations:** ^1^Department of Experimental Psychology, University of GhentGhent, Belgium; ^2^Department of Psychology, University of LiègeLiège, Belgium

**Keywords:** working memory, selective attention, short-term memory, long-term memory, executive function

Working memory refers to an intriguing and essential problem in cognitive science: How does the brain succeed in maintaining information for certain duration and perform mental operations on the stored information? An important avenue in tackling this issue stems from the relatively well-defined domain of selective attention. The role of selective attention in working memory has become increasingly dominant over the last decades as information stored in working memory has been shown to modulate (external) selective attention processing—and vice versa (for reviews see e.g., Awh and Jonides, 2001; Kiyonaga and Egner, 2013). Indeed, whereas attentional components in traditional working memory models typically relate to executive mechanisms assumed to control and supervise dedicated working memory buffers (e.g., Baddeley and Hitch, 1974; Baddeley, 2000), more recent models envisage working memory as directly and specifically emerging from selective attention turned to long-term memory representations (e.g., Cowan, 1999; Oberauer, 2009).

Despite the prominent role that selective attentional models of working memory play in current cognitive science, there is still much work to do in determining the precise implications of these models for specific aspects and characteristics of working memory—in both healthy and neuropsychological populations. The current Research Topic in Frontiers in Human Neuroscience aims to contribute to this challenge and offers novel empirical observations, state-of-the-art reviews, and intriguing theoretical proposals that illuminate the interplay between selective attention and working memory. In general, these contributions can be categorized into three broad classes of studies:

*First*, the majority of the papers involve empirical or theoretical efforts directly aimed at a better understanding of the interplay between selective attention and working memory. Camos and Barrouillet (2014) review the literature and identify attentional and non-attentional mechanisms that contribute to the maintenance of information in verbal working memory. Vergauwe and Cowan (2014) conclude on the basis of a review that attentional search within working memory happens at a high-speed processing rate of about 35–40 ms per item, and propose that this rate reflects the involvement of gamma oscillations in the brain. Pedale and Santangelo (2015) investigated to what degree sensory saliency of the item information during encoding modulates the current contents of WM during recollection. van Moorselaar et al. (2015) focus on the reverse relationship and investigate the time course in which information in working memory is protected against interfering visual input. Zokaei et al. (2014) tackle an outstanding question about the representational state of items in working memory that are not prioritized by attentional focusing. Abrahamse et al. (2014) hypothesize that serial order coding in verbal working memory appeals to the spatial attention system and thereby can be synthesized with selective attentional models on working memory in general. Ginsburg and Gevers (2015) conclude from their experiments that serial order in working memory and long-term memory both drive spatial effects but differentially so. De Fockert and Leiser (2014) tackle the relationship between working memory functioning and executive attention and wonder whether the lack of available working memory resources can in some situations enhance information processing. By reviewing the literature, Vandierendonck (2014) elaborates on the link between executive attention and selective attention and proposes a symbiotic model in which their close relationship is stressed. Relatedly, Kiyonaga and Egner (2014) investigate to what degree interactions between external attention and attention in working memory depend on shared attentional resources. Quak et al. (2015) extend these issues by emphasizing the need for a multisensory approach to working memory, and list a number of potential starting points.

*Second*, the study of the interplay between selective attention and working memory is not only fruitful for increasing our understanding of the cognitive underpinnings of working memory, it also helps us to achieve a better understanding of specific cognitive and behavioral difficulties across various types of populations. Holmes et al. (2014) demonstrate this to be the case for children with ADHD and children with low working memory capacity. In a similar vein Wong et al. (2015) show that this approach is also helpful to describe the subtle cognitive characteristics associated with specific genetic variations, such as the fragile X premutation. Roome et al. (2014) propose and show how the bisectioning of working memory into various (attentional and other) components can help to better understand the (normal) development of working memory.

*Third*, the current Research Topic also contains studies that extrapolate insights from the interplay between selective attention and working memory to other domains. For example, Meghanathan et al. (2015) investigate whether the availability of working memory resources can be predicted from oculometric parameters, such as eye fixation duration and pupil size. Moreover, Tanaka et al. (2014) use eye-tracking methodology to investigate to what degree individual differences in working memory capacity are predictive of word relocation processes during reading.

Overall, we are convinced that the studies in the current Research Topic provide much food for thought, as well as inspiration to keep up the empirical work on the interplay between selective attention and working memory. We would explicitly like to thank all the authors for their contributions as well as the reviewers for their critical reading.

## Conflict of interest statement

The authors declare that the research was conducted in the absence of any commercial or financial relationships that could be construed as a potential conflict of interest.
